# The Current Trend of the Translational Research Paradigm

**DOI:** 10.7759/cureus.2340

**Published:** 2018-03-19

**Authors:** Paul J Choi, R. Shane Tubbs, Rod J Oskouian

**Affiliations:** 1 Clinical Anatomy, Seattle Science Foundation; 2 Neurosurgery, Seattle Science Foundation; 3 Neurosurgery, Swedish Neuroscience Institute

**Keywords:** translational research, multi-disciplinary, bi-directionality, interdisciplinary training, community outreach, patient care

## Abstract

The translational research paradigm is a process of discovering basic science concepts and applying the knowledge in clinical practice, aiming to improve patient care. The stages involved in the paradigm form a complex network of shared knowledge amongst research collaborators, including patients. This nature of the paradigm allows those involved to work together effectively. However, the translational research paradigm is often overlooked by many scientists, educators, and research institutions. Hence, a large amount of comprehensive and hugely invested research projects fail to make a scientific impact. We aim to outline and describe this paradigm in order to aid in the successful translation of effective research.

## Introduction and background

The translational research paradigm depicts the stages involved in the process of discovering a concept from the basic science perspective and the implementation of such knowledge in clinical practice, aiming ultimately to yield public health improvements [1-3]. The stages are interwoven in a complex network of shared knowledge amongst research collaborators of various professions, including the patients [1-2]. This characteristic of the paradigm is referred to as bi-directionality and it allows those involved to work together effectively and ultimately translate an agreed, quality concept into clinical practice [1-2]. The paradigm is divided into four stages: T1, T2, T3, and T4 [1, 4]. This paper outlines and describes the paradigm and the current issues regarding this model to aid in successful translation of valuable research.

## Review

T1 Stage

The T1 stage is the preclinical aspect of translational research [1, 5]. In this stage, researchers identify a link between a basic science concept and human medicine [1, 6]. Once the link is verified, they demonstrate and simulate it in non-human models, tissue samples, or computer programs [1, 4]. The translation is also often carried out using healthy human volunteers [6]. T1 allows proof of concept to be established, i.e. the evidence of whether the concept is feasible [6]. It often involves Phase 1 clinical trials [6]. For instance, Peyraud et al. reported that a solid tumor responded to a CSF 1 inhibitor in-vivo [7].

T2 Stage

During the T2 stage, the concept established in T1 is implemented in human subjects so that its behavior in a human body can be examined under a controlled, ideal environment, i.e. a clinical trial [1, 6]. This allows the scientists to assess the efficacy or safety of an intervention [1]. Depending on the quality and validity of the evidence retrieved from such studies, the project can receive approval to establish an intervention for patient care [1, 5-6]. This stage involves Phase 2 and 3 clinical trials [6]. For instance, the National Cancer Institute had a Phase 2 clinical trial numbered 15-C-0093 which assessed the efficacy of PLX3397 (Pexidarinib), a CSF1 receptor inhibitor in children and adolescents with inoperable refractory solid tumors such as rhabdomyosarcoma of the head and neck region (https://clinicaltrials.gov/ct2/show/NCT02390752).

T3 Stage

T3 is the stage during which the confirmed evidence from human subjects is introduced and disseminated into a patient care setting [1, 2, 4-6]. The aim is not only to treat patients with existing diseases, but also to help confirm or disprove the results of the clinical trials conducted in the previous stages [1]. The evaluative nature of this stage can introduce further research topics to discuss new clinical concepts and incompletely understood clinical outcomes [1, 6]. T3 involves a Phase 4 clinical trial [6].  For instance, the PLX3397 study was unfortunately suspended temporarily because of a request by the FDA owing to safety concerns and could not progress to a Phase 3 or 4 trial.

T4 Stage

T4 is the ultimate translation stage [1]. The positive outcomes stemming from the topic, which were studied in the previous stages, are reproduced at a population level to establish its validity and value [1, 4-5]. This stage assesses the effects of the newly-established intervention and can lead to the beginning of a new project to study and minimize possible weaknesses in the currently developed intervention [1]. A successful translation leads to improved public health [6, 8]. In this stage of the paradigm, the cost of the intervention is also studied [5]. The ultimate goal of this stage is to maximize public health and minimize the financial burden the intervention could impose [4].

Obstacles in Translational Research

Although the translational paradigm appears well-constructed and highly systematic, it is not without flaws. Several factors can impair the translation process, e.g., failure to establish some of its core aspects such as developing a focused topic of interest, carrying out an effective multidisciplinary effort, using appropriate Biobanking standard protocols, establishing and maintaining bi-directionality, installing deep education and mentoring systems, and designing community outreach programs [2, 8-11]. Among these, establishing a good interaction amongst the research collaborators to educate each other and share knowledge seems to be the “hot topic” in today’s medical literature, since the United States has very few skills training and education programs in place to support the multidisciplinary, bi-directional nature of translation research [2]. This scarcity of effective educational programs has led to an unsatisfactory intervention approval rate (failure rate 95%) despite increased investment and significant advances in molecular science during the past two decades [8, 10, 12].

For instance, Burns et al. emphasize failure to establish effective research training and dedicated mentorship programs as the main obstacle to translational research [9]. Gonzales et al. define interplay of the members of the community, community groups, healthcare professionals, and government agencies as key to a successful translation [2, 11]. They also emphasize the importance of involving trainees of various professions in the education and mentoring programs, highlighting the importance of implementing interdisciplinary efforts within the education system to develop and learn additional skills, i.e. multidisciplinary teamwork, ultimately yielding competent physician and scientist researchers who work efficiently [2-3, 9, 11, 13]. Moreover, Knowlton et al. stress that there are too few skilled researchers in translational research owing to lack of effective communication among researchers of different educational and professional backgrounds [12].

In fact, effective interdisciplinary collaboration and education/training programs are the foundation of all other aspects of a successful translational research mentioned previously, i.e. study design, development of robust Biobanking protocols, establishment of a successful community outreach program, and maintaining bi-directional flow [8]. Many research institutions in the United States have now introduced survey evaluations to improve understanding of their current teamwork as a multidisciplinary group [13].

## Conclusions

As can be seen, translational research has multiple stages that form a complex network within which the elements build upon each other. It flows well, bi-directionally, only when an effective education system stressing the importance of teamwork, and a robust multidisciplinary effort integrated within the education system, can yield well-rounded and competent researchers who can ultimately commit to improved community health and healthcare costs.

## References

[1] (2018). Translational science spectrum. https://ncats.nih.gov/translation/spectrum.

[2] Gonzales R, Handley MA, Ackerman S (2012). A framework for training health professionals in implementation and dissemination science. Acad Med.

[3] Dilmore TC, Moore DW, Bjork Z (2013). Developing a competency-based educational structure within clinical and translational science. Clin Transl Sci.

[4] Munoz DA, Nembhard HB, Kraschnewski JL (2014). Quantifying complexity in translational research: an integrated approach. Int J Health Care Qual Assur.

[5] Griswold-Theodorson S, Ponnuru S, Dong C (2015). Beyond the simulation laboratory: a realist synthesis review of clinical outcomes of simulation-based mastery learning. Acad Med.

[6] (2018). Clinical and translational research spectrum. https://catalyst.harvard.edu/pathfinder/.

[7] Peyraud F, Cousin S, Italiano A (2017). CSF-1R Inhibitor Development: Current Clinical Status. Curr Oncol Rep.

[8] Searles A, Doran C (2016). An approach to measuring and encouraging research translation and research impact. Health Res Policy Syst.

[9] Burns LJ, Clayton CP, George JN, Mitchell BS, Gitlin SD (2015). The effect of an intense mentoring program on junior investigators' preparation for a patient-oriented clinical research career. Acad Med.

[10] Collins FS (2011). Reengineering translational science: the time is right. Sci Transl Med.

[11] Scott CS, Nagasawa PR, Abernethy NF (2014). Expanding assessments of translational research programs: supplementing metrics with value judgments. Eval Health Prof.

[12] Knowlton AA, Rainwater JA, Chiamvimonvat N (2013). Training the translational research teams of the future: UC Davis-HHMI Integrating Medicine into Basic Science program. Clin Transl Sci.

[13] Rubio DM (2013). Common metrics to assess the efficiency of clinical research. Eval Health Prof.

